# Diagnostic evaluation of people with hypertension in low income country: cohort study of “essential” method of risk stratification

**DOI:** 10.1136/bmj.a1387

**Published:** 2008-09-19

**Authors:** Gregorio Montalvo, Fausto Avanzini, Mariella Anselmi, Rosanna Prandi, Samuel Ibarra, Monica Marquez, Daniela Armani, Juan-Martín Moreira, Cynthia Caicedo, Maria Carla Roncaglioni, Fabio Colombo, Paola Camisasca, Valentina Milani, Simon Quimì, Felix Gonzabay, Gianni Tognoni

**Affiliations:** 1 Centro de Epidemiología Comunitaria y Medicina Tropical (CECOMET), Esmeraldas, Ecuador; 2 Laboratorio di Ricerca in Medicina Generale, Istituto di Ricerche Farmacologiche Mario Negri, 20156 Milan, Italy; 3 Centro Malattie Tropicali, Ospedale S. Cuore, 37034 Negrar, Verona, Italy; 4 Sistema Local de Salud, Ministerio de Salud Pública, Borbón, Ecuador; 5 Laboratorio di Statistica Medica, Istituto di Ricerche Farmacologiche Mario Negri, Milan; 6 Consorzio Mario Negri Sud, 66030 Santa Maria Imbaro, Chieti, Italy

## Abstract

**Objectives** To explore the predictive power of a risk stratification method for people with hypertension based on “essential” procedures (that is, available in economically less developed areas of the world), comparing it in the same population with the results given by the method suggested by the 1999 World Health Organization-International Society of Hypertension (WHO-ISH) guidelines.

**Design** Prospective cohort study of outcomes according to cardiovascular risk profile at baseline.

**Setting** Primary care in a poor rural area of the Ecuadorian forest.

**Participants** 504 people with hypertension prospectively monitored for a mean of 6.7 (SD 2.3) years.

**Interventions** Essential data included blood pressure, medical history, smoking, age, sex, and diagnosis of diabetes; the WHO-ISH methods additionally included measurement of fasting blood glucose, total cholesterol, and creatinine, urinalysis, and electrocardiography.

**Main outcome measures** Cardiovascular events and total deaths.

**Results** With both methods there was a highly significant association between the level of predicted risk and the incidence of cardiovascular events and of total deaths: up to three quarters of all cardiovascular events and two thirds of all deaths were reported among people classified as at high or very high risk with either method. The predictive discrimination of the essential method is comparable with the WHO-ISH with C statistics (95% confidence interval) of 0.788 (0.721 to 0.855) and 0.744 (0.673 to 0.815), respectively, for cardiovascular events and 0.747 (0.678 to 0.816) and 0.705 (0.632 to 0.778) for total mortality.

**Conclusions** The risk stratification of patients with hypertension with an essential package of variables (that is, available and practicable even in the economically less developed areas of the world) serves at least as well as the more comprehensive method proposed by WHO-ISH.

## Introduction

There is increasing emphasis in the major general and specialised scientific journals on the burden of cardiovascular diseases in terms of mortality and morbidity and of hypertension as a leading risk factor in low income countries.^1^ ^2^ ^3^ ^4^ ^5^ ^6^ ^7^ ^8^ ^9^ The instruments and strategies proposed^10^ ^11^ ^12^ ^13^ ^14^ to deal with these problems, however, derive mostly from experimental and observational studies produced in more developed countries.

Studies like INTERHEART provide important support for the idea that the components of cardiovascular risk are substantially comparable across a broad spectrum of populations and healthcare systems.^15^ ^16^ Cohort studies in low income countries, aiming to assess not only the causal side of the risk but the critical question of the transferability of measures recommended to identify patients at risk and to influence their clinical outcomes, are still rare.^17^

This situation is likely to produce an important cultural distortion in the perception and management of problems by local and international health professionals and planners, further aggravating the impact of well known socioeconomic inequalities.^17^ ^18^ Countries with restricted resources need a cost effective cardiovascular preventive strategy^19^ so that candidates for preventive interventions can be stratified by absolute level of cardiovascular risk and priority given to those at higher risk of complications.^20^ ^21^ ^22^

We explored the predictive power of a risk stratification method for hypertension based on “essential” procedures (that is, available in good community practice even in the economically less developed areas of the world), comparing it in the same population with the results given by the method suggested by the World Health Organization-International Society of Hypertension (WHO-ISH) guidelines^11^ in an area that could be described as a model of epidemiological transition^8^ and where hypertension has been carefully documented as the major component of a high cardiovascular risk profile.^23^

## Methods

The health district of Borbón in Ecuador is an area of about 5000 km^2^ almost completely covered by equatorial forest. The population of about 25 000 people, 85% black, 10% Amerindian Indios, and 5% white, is scattered in 129 villages along three rivers, which often serve as the only transport routes through the area; 84% of the population is classified as poor and 34% extremely poor, and one third of the adults are illiterate. The area is served by one hospital with 20 beds in Borbón, 12 health centres along the rivers managed by non-specialised nursing staff, and a network of 50 voluntary health “promotors” (“promotores de salud”), with the occasional supervision from rural medical doctors. Monthly meetings of all the district health team workers are called regularly to allow close and participatory monitoring of the quality of delivered care, with analysis of all relevant clinical events.

### Screening for hypertension and diagnostic investigation

Between 1995 and 2001 a screening programme of the population aged 18 and over was set up to assess the size and impact of the risk of hypertension. Rural medical doctors visited villages and measured blood pressure with calibrated aneroid sphygmomanometers in seated patients. They recorded the lower of two values measured 5 minutes apart, rounded to the nearest 5 mm Hg. All participants with systolic blood pressure ≥140 mm Hg or diastolic ≥90 mm Hg were rechecked the next day. The results of this screening programme have been reported elsewhere.^23^

The cohort of 1643 people with hypertension (systolic blood pressure ≥140 mm Hg or diastolic ≥90 mm Hg, or both, at the screening and the next day, or taking antihypertensive drugs) was prospectively monitored, and all causes of death and major cardiovascular events (stroke, transient ischaemic attack, myocardial infarction, heart or renal failure, and vascular disease) were recorded. The rural medical doctors diagnosed non-lethal cardiovascular events during their periodic visits to the communities. All deaths were included in a registry based on an immediate postmortem form filled in by the local nurse or health promoter. The rural medical doctors subsequently defined cause of deaths with verbal autopsies.^24^ ^25^ ^26^

With the resources provided by an international donation, between 1998 and 2001 a subset of participants with hypertension underwent all the laboratory and instrumental investigations recommended by the World Health Organization (WHO) and the International Society of Hypertension (ISH).^10^ ^11^ Tests included fasting blood glucose, total cholesterol, and creatinine concentrations; urinalysis; and electrocardiography. The local hospital laboratory could not measure plasma potassium concentrations. Participants in the subset lived in the more accessible villages because, in the absence of electricity, blood samples had to be stored in a portable refrigerator and had to be transferred as soon as possible to the local hospital at Borbón. Complete laboratory data were available for 504 of the 714 participants evaluated.

The results of the laboratory tests, with clinical history, physical examination, and blood pressure, served to estimate each participant’s future absolute risk of major cardiovascular events, as suggested by WHO-ISH guidelines.^11^ These estimates are based on blood pressure and the presence of other risk factors and history of diseases (age, sex, family history of premature cardiovascular disease, smoking, cholesterol, diabetes, target organ damage, associated clinical conditions) (fig 1). The rural medical doctors diagnosed associated clinical conditions (cerebrovascular or coronary diseases, heart and renal failure, vascular disease) on the basis of clinical history, physical examination, and, when available, clinical record, as suggested by the WHO-ISH 1999 guidelines.^11^ We could not evaluate hypertensive retinopathy because of the lack of equipment and technical competence, and a family history of premature cardiovascular disease could not be assumed to be retrievable information. This should not have altered the predictive power of the WHO-ISH method as generalised or focal narrowing of the retinal arteries has low specificity^27^ and has therefore been dropped as target organ damage in the 2003 WHO-ISH guidelines,^28^ and advanced retinopathy is usually associated with severe hypertension, that is in itself a high risk condition. Moreover, the prevalence of a family history of premature cardiovascular disease in an adult population in the transition phase is expected to be low.

**Figure fig1:**
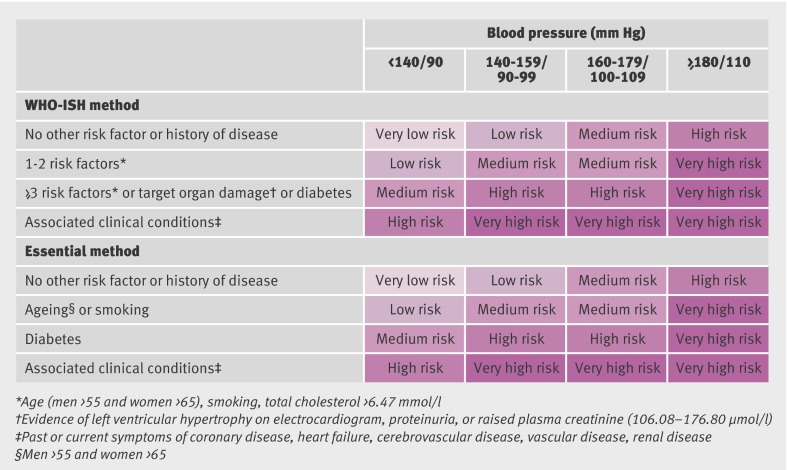
**Fig 1** Stratification of cardiovascular risk to quantify prognosis: WHO-ISH and essential methods

As the 1999 WHO-ISH guidelines do not suggest specific electrocardiographic criteria for left ventricular hypertrophy, we adopted the Framingham criterion for left ventricular hypertrophy as providing a better score of predictive power.^29^ We modified the original WHO-ISH stratification table to include all those with known hypertension, including those taking treatment who had normal blood pressure readings on the day of the laboratory test (fig 1).[Fig fig1]

As the laboratory and instrumental investigations recommended by the WHO-ISH are not usually available for people living in this poor region of the equatorial forest, in the same population evaluated according to the method suggested by WHO-ISH guidelines we studied the predictive power of a simplified risk stratification method based only on the data available even in this economically underdeveloped area. In addition to blood pressure, this essential method includes age, smoking, diabetes (which in this population is usually self diagnosed by tasting urine), and associated clinical conditions (fig 1).[Fig fig1]

We compared the two risk prediction methods using cardiovascular events (the first non-lethal cardiovascular event or cardiovascular death) as the primary outcome and total mortality as secondary outcome.

### Statistical analysis

Descriptive data are expressed as counts (percentages) for categorical data and as means (SD) for continuous variables, as appropriate. We measured concordance between the two methods in individual risk stratification by the weighted κ statistic. Differences in the rate of events according to risk categories were evaluated with the Mantel-Haenszel test for linear association. Plots of the Kaplan-Meier estimate of the survival curves according to the cardiovascular risk categories of the two methods are shown for cardiovascular events and total deaths. Survival plots run to the mean follow-up of seven years. We constructed two multivariable Cox proportional hazards models for cardiovascular events for each method, adjusting for four classes of blood pressure and four categories of other risk factors and history of disease (fig 1).[Fig fig1] To compare the predictivity of the two stratification methods we used receiver operating characteristic (ROC) curves based on the same variables as the Cox regression models. These curves are the plot of the true positive rate (sensitivity) in relation to the false positive rate (100−specificity).^30^ The area under the curve, which is equivalent to the C statistic, provides a summary measure of the accuracy of the diagnostic test, which can also be thought of as how well the test distinguished between those with and without outcomes (cardiovascular events or death). The curves were calculated up to a maximum of seven years. To assess the diagnostic performance of the two methods of risk stratification we compared predicted cardiovascular risk (medium and over, high and over, or very high) with observed outcomes (incidence of cardiovascular events and total deaths during follow-up), calculating the sensitivity and specificity. All statistical analyses were done with SAS (version 9), and all tests were done at the 5% significance level.

## Results

Table 1 shows the main characteristics at baseline of the 504 participants with hypertension evaluated according to both methods. Most had known about their hypertension for many years (5-10 years for 172 (34%) and >10 years for 119 (24%)). Only 82 patients (16%) were being treated with antihypertensive drugs, and 150 (30%) had received advice on non-pharmacological measures (such as reduction in salt intake). Of the 504 patients, 86% had blood pressure ≥140/90 mm Hg and 34% had blood pressure ≥180/110 mm Hg. Fifty six (11%) had evidence of target organ damage (evidence of left ventricular hypertrophy on the electrocardiogram, proteinuria, or slightly raised plasma creatinine concentrations). Associated clinical conditions (history or current symptoms of coronary disease, heart failure, cerebrovascular disease, vascular disease, renal disease) were reported in 22 (4%), mainly cerebrovascular events (n=14) and heart failure (n=6).

**Table 1 tbl1:** Main clinical characteristics at baseline of 504 people with hypertension investigated with WHO-ISH and essential prognostic stratification methods. Figures are numbers (percentage) of participants unless stated otherwise

	Data
Age (years):	
Mean (SD)	55.5 (14.4)
>55 for men or >65 for women	164 (33)
Sex (female)	337 (67)
Race (black)	470 (93)
Systolic and diastolic blood pressure (mm Hg):	
<140/90	72 (14)
140-159/90-99	126 (25)
160-179/100-109	133 (27)
≥180/110	173 (34)
Mean (SD)	159.7 (31.1)/97.4 (15.3)
Current smoking	35 (7)
Total cholesterol >6.47 mmol/l	45 (9)
Proteinuria	10 (2)
Raised plasma creatinine (106.08-176.80 µmol/l)	37 (7)
Evidence of left ventricular hypertrophy on ECG	12 (2)
Diabetes mellitus	30 (6)
Cerebrovascular disease	14 (3)
Coronary disease	2 (0.4)
Heart failure	6 (1)
Renal failure	1 (0.2)
Vascular disease	0

ECG=electrocardiogram.

### Stratification by absolute level of cardiovascular risk

Table 2 shows the distribution of participants according to cardiovascular risk factors other than blood pressure, target organ damage, and associated clinical conditions, evaluated with and without laboratory investigations. As expected, laboratory investigations increased the proportion of participants identified with three or more associated cardiovascular risk factors, target organ damage, or diabetes. In 433 patients (86%), however, the two methods were concordant in weighting the “other risk factors and disease history” with a weighted κ value of 0.764.

**Table 2 tbl2:** Risk factors* (other than blood pressure) and cardiovascular risk estimated with and without laboratory investigations†. Figures are numbers (percentage) of participants

	With laboratory investigations	Without laboratory investigations
No other risk factors	235 (47)	285 (57)
1-2 risk factors	167 (33)	168 (33)
≥3 risk factors or TOD‡ or diabetes	80 (16)	29 (6)
Associated clinical conditions§	22 (4)	22 (4)
Stratification of total cardiovascular risk:		
Very low risk	33 (6)	47 (9)
Low risk	94 (19)	99 (20)
Medium risk	160 (32)	157 (31)
High risk	102 (20)	104 (21)
Very high risk	115 (23)	97 (19)

*Age (men >55 and women >65), current smoking, total cholesterol >6.47 mmol/l.

†Electrocardiography, serum total cholesterol, serum creatinine, urinalysis for protein.

‡Target organ damage (TOD) included evidence of left ventricular hypertrophy on electrocardiogram, proteinuria, raised plasma creatinine (106.08-176.80 µmol/l).

§History or current symptoms of coronary disease, heart failure, cerebrovascular disease, vascular disease, renal disease.

As expected, laboratory results identified a larger proportion of participants classified at higher risk (table 2).[Table tbl2] In 450 (89%), however, the two methods agreed in stratifying total cardiovascular risk with a weighted κ value of 0.902 (table 3). In only 16 patients out of 217 (7%) did the essential method not confirm the high or very high risk defined by the WHO-ISH method (table 3).[Table tbl3]

**Table 3 tbl3:** Concordance* between WHO-ISH and essential prognostic stratification methods in defining individual global cardiovascular risk

Cardiovascular risk according to essential method	Cardiovascular risk according to WHO-ISH methods					Total
	Very low	Low	Medium	High	Very high	
Very low	33	5	9	0	0	47
Low	0	89	6	4	0	99
Medium	0	0	145	12	0	157
High	0	0	0	86	18	104
Very high	0	0	0	0	97	97
Total	33	94	160	102	115	504

*Weighted κ=0.9023.

### Incidence of cardiovascular events during follow-up according to risk prediction

On 31 December 2007 we examined the rates of cardiovascular events and total deaths for all 504 patients with hypertension. During a mean follow-up of 6.7 (SD 2.3) years (range 12 days-9.7 years), 76 (15%) had a cardiovascular event and 74 (15%) died. Thirty two had one or more strokes (19 fatal), 30 had one or more episodes of heart failure (18 fatal), 14 had one or more transient ischaemic attacks, four died suddenly, three had an acute myocardial infarction (two fatal), and one had fatal renal failure. Fourteen died from non-cardiovascular causes and 16 of unknown causes.

The proportion of participants with cardiovascular events was significantly associated with baseline blood pressure: respectively 7%, 11%, 10%, and 25% in those with normal blood pressure (<140/90 mm Hg), mild (140-159/90-99 mm Hg), moderate (160-179/100-109 mm Hg), and severe (≥180/110 mm Hg) hypertension (P<0.001 for trend). The proportion with cardiovascular events was also significantly associated with the four categories of other risk factors and history of disease considered in the WHO-ISH method (7%, 19%, 15%, and 68%, P<0.001 for trend) and the essential method (6%, 21%, 24%, and 68%, P<0.001 for trend).

Multivariate Cox analyses confirmed that in this population the criteria adopted by both methods (blood pressure classes and categories of other risk factors and disease history) were significantly associated with the incidence of cardiovascular events (data not shown). Kaplan Meier survival curves in patients at very low, low, medium, high, and very high cardiovascular risk according to both methods indicated a highly significant association between the level of predicted risk with both methods and the incidence of cardiovascular events (log rank test, P<0.001) (fig 2).[Fig fig2] The ROC curves show that the predictive discrimination of the essential method was comparable with that of the WHO-ISH method with C statistics 0.788 (95% confidence interval 0.721 to 0.855) and 0.744 (0.673 to 0.815), respectively (fig 3).[Fig fig3]

**Figure fig2:**
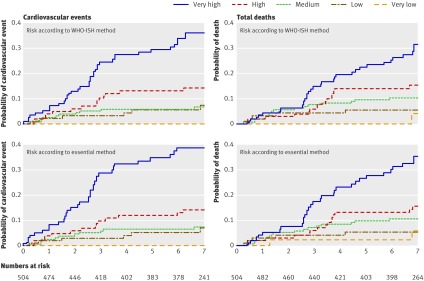
**Fig 2** Kaplan-Meier survival curves for cardiovascular events (first non-lethal cardiovascular event or cardiovascular death) and for total deaths according to cardiovascular risk categories of WHO-ISH and essential methods

**Figure fig3:**
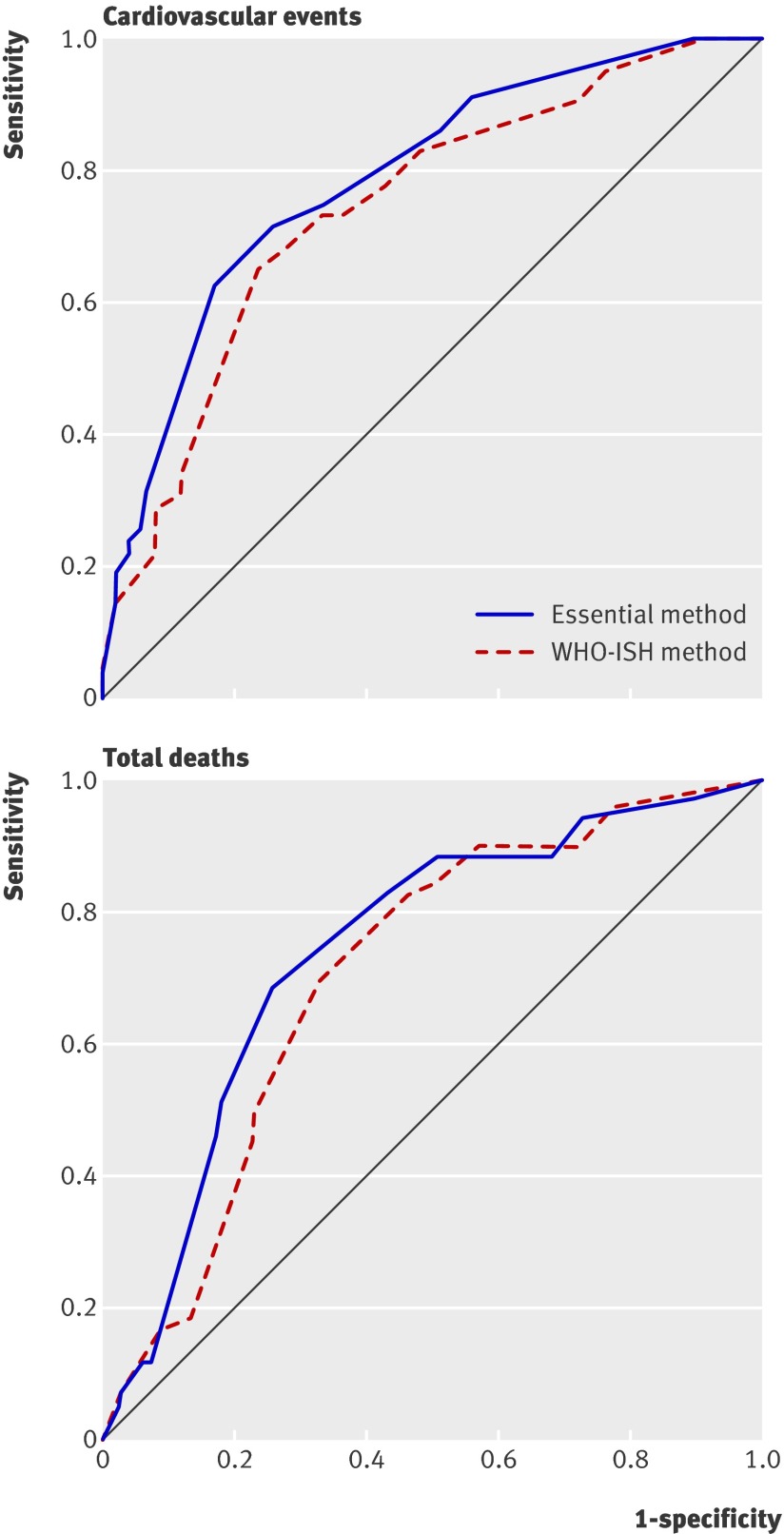
**Fig 3** ROC curves for prediction of cardiovascular events and of total deaths according to WHO-ISH and essential methods

Table 4 shows the sensitivity and specificity of both methods against different risk thresholds for cardiovascular events.[Table tbl4] There were no significant differences between the two methods at any risk threshold for all the criteria.

**Table 4 tbl4:** Sensitivity and specificity (95% confidence interval) of two stratification methods at various cardiovascular risk thresholds

	Risk level		
	≥ Medium	≥ High	Very high
**Cardiovascular events**			
Sensitivity:			
WHO-ISH	91.0 (84.2 to 97.9)	76.1 (65.9 to 86.3)	55.2 (43.3 to 67.1)
Essential	91.0 (84.2 to 97.9)	74.6 (64.2 to 85.0)	53.7 (41.8 to 65.7)
Specificity:			
WHO-ISH	27.7 (23.5 to 31.9)	62.0 (57.5 to 66.6)	82.2 (78.6 to 85.7)
Essential	32.0 (27.7 to 36.4)	65.5 (61.0 to 69.9)	86.0 (82.8 to 89.3)
**Total deaths**			
Sensitivity:			
WHO-ISH	90.0 (83.0 to 97.0)	67.2 (56.1 to 78.1)	45.7 (34.0 to 57.4)
Essential	88.6 (81.1 to 96.0)	65.7 (54.6 to 76.8)	44.3 (32.7 to 55.9)
Specificity:			
WHO-ISH	27.7 (23.4 to 31.9)	60.8 (56.2 to 65.4)	80.9 (77.2 to 84.6)
Essential	31.8 (27.4 to 36.2)	64.3 (59.8 to 68.8)	84.8 (81.4 to 88.2)

Up to three quarters of all participants with cardiovascular events were classified as at high or very high risk under either stratification methods: 57 out of 76 (75%) among the 217 identified at risk with the WHO-ISH criteria and 56 out of 76 (74%) among the 201 identified at risk with the essential package.

Only two of the 76 cardiovascular events were in participants who were classified according to the essential method as at lower risk than according to the WHO-ISH method: one was a patient classified medium instead of high risk and the other was classified as high risk instead of very high.

The results did not change substantially when we restricted the analyses to the 357 patients with blood pressure ≥140/90 mm Hg who were not taking antihypertensive drugs at baseline. The predictive discrimination of the essential method was comparable with that of the WHO-ISH, with C statistics 0.759 (0.667 to 0.851) and 0.715 (0.619 to 0.811), respectively.

The use of electrocardiographic criteria for the detection of left ventricular hypertrophy by a more sensitive method than the Framingham criterion, such as the Romhilt-Estes and Perugia score or the Sokolow-Lyon and Cornell voltages,^31^ would reduce the predictive discrimination of the WHO-ISH method: C statistics 0.739 (0.668 to 0.810), 0.725 (0.652 to 0.798), 0.724 (0.651 to 0.797), and 0.723 (0.650 to 0.796), respectively.

### Total mortality during follow-up according to risk prediction

The percentages of all deaths in patients at very low, low, medium, high, and very high cardiovascular risk were 3%, 6%, 10%, 16%, and 30% according to the WHO-ISH method and 4%, 6%, 10%, 15%, and 35% according to the essential method. As for cardiovascular events, even with total deaths as outcome, both stratification methods showed a significant association between the level of predicted risk and mortality (log rank test, P<0.001) (fig 2); similar predictive discrimination with C statistic 0.705 (0.632 to 0.778) for the WHO-ISH method and 0.747 (0.678 to 0.816) for the essential method (fig 3); and comparable sensitivity and specificity (table 4).[Table tbl4]

## Discussion

A simplified method for risk stratification of patients with hypertension based on variables that can be classified as essential (because of their affordability, applicability, and reliability even in the economically less developed areas of the world) performs at least as well as the more comprehensive method recommended by WHO-ISH guidelines.^11^ Among the high risk patients identified without any laboratory or instrumental examination we recorded three quarters of all the cardiovascular events occurring during a seven year follow-up (sensitivity 75% *v* 76% for the WHO-ISH method). The specificity of the simplified method was also close to that of the WHO-ISH criteria.

Hypertension is increasingly recognised as a major cause of mortality and morbidity in low income countries, where its complications arise at an earlier age than in developed countries.^6^ ^7^ ^8^ ^9^ The most cost effective and nowadays universally recommended approach for the treatment of hypertension should be based on the absolute risk of cardiovascular disease.^7^ ^10^ ^11^ Methods for the identification of high risk patients should be simple, reproducible, easily accessible, and low cost, especially in developing countries.^19^ The scheme for stratifying the global risk in hypertension was formulated by WHO-ISH in 1999^11^ and subsequently updated and revised.^28^ ^32^ No data are available, however, on the real applicability of this method in developing countries.

### Strengths and limitations

Our findings have the advantage of reflecting real life conditions, where the clinical outcomes include all the relevant field variables that can arise over a follow-up that was particularly long for a difficult and deprived setting of life and care, although we might have underestimated the overall rate of cardiovascular events because of the difficulties of doing instrumental and laboratory tests in this setting. A better classification of cardiovascular events, however, should not have influenced the results of the comparison between the two risk stratification approaches. Also, to overcome this possible limitation, we included total deaths in the evaluation of the prognostic power of the two methods. 

In a remote rural region of a low income country the WHO-ISH criteria would be hard to apply in everyday practice.^19^ Some of the variables could not be measured either because of the lack of equipment or technical skills (for example, examination of the optic fundus) or the unavailability or unreliability of information (family history of premature cardiovascular events). In our study we were able to carry out systematic electrocardiography and biochemical analyses because of an ad hoc research grant that helped to overcome logistic and organisational barriers. In routine practice people with hypertension should attend the nearest local hospital to be examined, which might require a full day of difficult travel (such as river navigation), affordable by only a minority of people. In regions with limited medical staff the simplified approach for first line screening of people at higher risk could be easily used by non-medical staff. The applicability of this method by the “promotores de salud” is currently being assessed in the health district of Borbón.

### Comparison with other studies

Though we know of no other similar studies, the problems described here are likely to be representative of the logistic and economic barriers in many other rural areas of Latin America and many other low income countries. Moreover, although our study population was mainly made up of black people with high and often untreated hypertension, the results in terms of feasibility and predictive accuracy of the proposed simplified method for stratifying cardiovascular risk should be easily transferable and applicable in other settings at a similar stage of the epidemiological transition.^8^ ^9^

### Implications

Operationally, the resources needed for the procedures included in the “standards” could be drastically reduced, thus allowing broader coverage of the population as well as closer care of those at highest risk or those already disabled. For example, nowadays in the district of Borbón the cost to a patient for the laboratory tests recommended by WHO-ISH is equivalent to the cost of almost two years’ treatment with antihypertensive drugs.

From the public health point of view, our data do not support the consolidated idea of a direct proportion between more sophisticated and costly approaches and better care. While the best existing knowledge must certainly be kept in mind, the approach we adopted highlights the priority and the need to put the issue of practicable care in the forefront. Time should not be spent nor resources invested in reiterating general recommendations but in the field, assessing the outcomes of due and possible care and producing original knowledge of the degree of effectiveness (or epidemiological efficacy) of essential practices (that is, recommended and transferable).

Thirty years ago, the WHO report on essential drugs^33^ was conceived as a tool to assure that most people could have access to drugs as part of their rights to life. Governing bodies were challenged to transform the essential drugs list into concrete health policies. We now also need essential prognostic tools, but there are few field projects that prospectively monitor the outcomes of affordable (not simply recommended) diagnostic practices.

**What is already known on this topic**

The critical role of arterial hypertension in the increasing burden of cardiovascular diseases in economically less developed areas of the world is clearly recognised, but it is usually addressed on the basis of data and strategies reflecting findings and projections produced in contexts that make them hardly transferable to the real settings of low income countries

**What this study adds**

The risk stratification of hypertensive patients with an “essential” package of variables (that is, available and practicable even in the economically less developed areas of the world) serves at least as well as a more comprehensive method with laboratory and instrumental investigations

## Notes

We thank Mauricio Espinel and Javier Corrales, medical directors of the health district of Borbón during the screening; the medical team of the health district (Bolivar Jalca, Javier Zambrano); the nurses of the district (Margarita Padilla, Daniel Tiller, Alba Chumo, Neida Mina, Juana Valencia, Hilda Guerrero, María Quiñonez, Angel Añapa, Demetrio Tapuyo, Elsa Arroyo, Lindon Corozo, Feliza Caicedo, Magda Lisley Corozo, Veronica Borja); the network of the voluntary health promotors “Asociaciòn de promotores de salud del area Borbón-APSA” (Heroína Arboleda, Sobeida Arroyo, Oberliza Caicedo, Gonzalo Medina, Amelia Preciado, Santo Mina, Adalín Valencia, Jorge Peralta, María Corozo, Ramona Sabando, Lucrecia Palacios, Erenni Cuero, Blanca Vega, Estela Arroyo, Marcos Borja, Juliana Mina, Pastor Mercado, María Arroyo, Julio Valdez, María Ayoví, Carmen Ayoví, Verónica Borja, Heriberto De la Cruz, Màrtires Ortiz, Hernán Tapuyo, Pedro Añapa, Pedro Luis Añapa, Edgar Añapa); and the local people who participated with enthusiasm in the programmes. We also thank Simona Barlera for statistical support and Fiorenza Clerici, Angela Palumbo, and Guya Sgaroni for secretarial assistance. English editing was kindly done by J D Baggott.

Contributors: GM, FA, MA, RP, J-MM, MCR, and GT were responsible for planning the study. GM, FA, and MCR prepared the first draft of the paper, and GT and MA revised it critically for important intellectual content. PC, GM, MA, RP, SI, MM, DA, J-MM, CC, SQ, and FG were responsible for data collection and development of the dataset. FC and VM carried out the statistical analyses. All authors contributed to the interpretation of the results, and approved the final version of the paper. GT is guarantor.

Ethical approval: Not required.

Provenance and peer review: Not commissioned; externally peer reviewed.

## Notes

**Cite this as:** *BMJ* 2008;337:a1387
